# Intraductal papillary-mucinous carcinoma with portal vein tumor thrombus and multifocal liver metastasis: An autopsy case

**DOI:** 10.3892/mco.2021.2263

**Published:** 2021-03-13

**Authors:** Naohiro Matsushita, Akitoshi Douhara, Hirotsugu Ueno, Shohei Asada, Koji Murata, Koji Yanase, Masahiro Tsutsumi

**Affiliations:** 1 Clinical Study and Training Center, Saiseikai Chuwa Hospital, Sakurai, Nara 633-0054, Japan; 2 Department of Gastroenterology, Saiseikai Chuwa Hospital, Sakurai, Nara 633-0054, Japan; 3 Department of Pathology, Saiseikai Chuwa Hospital, Sakurai, Nara 633-0054, Japan

**Keywords:** intraductal papillary-mucinous carcinoma, portal invasion, portal vein tumor thrombosis, liver metastasis

## Abstract

The prognosis of intraductal papillary-mucinous neoplasm is superior to that of conventional pancreatic ductal adenocarcinoma. Only a few advanced cases of intraductal papillary-mucinous carcinoma (IPMC) have been reported to date. We herein report the case of a 78-year-old male patient with advanced pancreatobiliary type IPMC with portal vein invasion and liver metastasis. The IPMC invaded the portal vein to form a tumor thrombus and it also metastasized to the liver via the portal vein. After receiving best supportive care, the patient succumbed to the disease following an exacerbation of IPMC 90 days after the initial presentation. On autopsy, a very long tumor thrombus was identified, along with liver metastatic lesions, which had retained the structure of the primary IPMC on histological examination.

## Introduction

Intraductal papillary-mucinous neoplasm (IPMN) of the pancreas is a cystic tumor arising from the cells lining the pancreatic ducts. IPMN is divided into main-duct, branch-duct and mxixed-type lesions, depending on the site of origin (1). The disease spectrum ranges from low-grade dysplasia to invasive intraductal papillary-mucinous carcinoma (IPMC) (2). Moreover, IPMN may occasionally be associated with pancreatic carcinoma at a site distant from IPMN (1). Histologically, IPMNs are subdivided into intestinal, pancreatobiliary, gastric and oncocytic types based on the expression of immunohistochemical markers, such as mucin core protein (MUC)1, MUC2 and CDX2(3). Generally, the prognosis following surgical resection of IPMN with an associated invasive carcinoma has been reported to be superior to that of pancreatic ductal adenocarcinoma (PDAC) (4). However, the prognosis of the pancreatobiliary type IPMN is worse compared with that of the other subtypes (5). We herein report a case of a pancreatobiliary type IPMC with formation of a portal vein tumor thrombus and multifocal liver metastasis.

## Case report

A 78-year-old man visited a regional hospital with complaints of fever and vomiting. An abdominal plain computed tomography (CT) scan at the hospital revealed dilatation of the bile ducts and the presence of cystic lesions in the pancreatic head and liver, and the patient was referred to Saiseikai Chuwa Hospital (Sakurai, Japan) for further examination and treatment.

The patient had a history of hypertension and benign prostatic hyperplasia and received regular treatment with nifedipine and tamsulosin hydrochloride. There was no history of liver dysfunction or a specific family history. The patient reported no allergies, cigarette smoking, or alcohol consumption.

**Figure 1:**
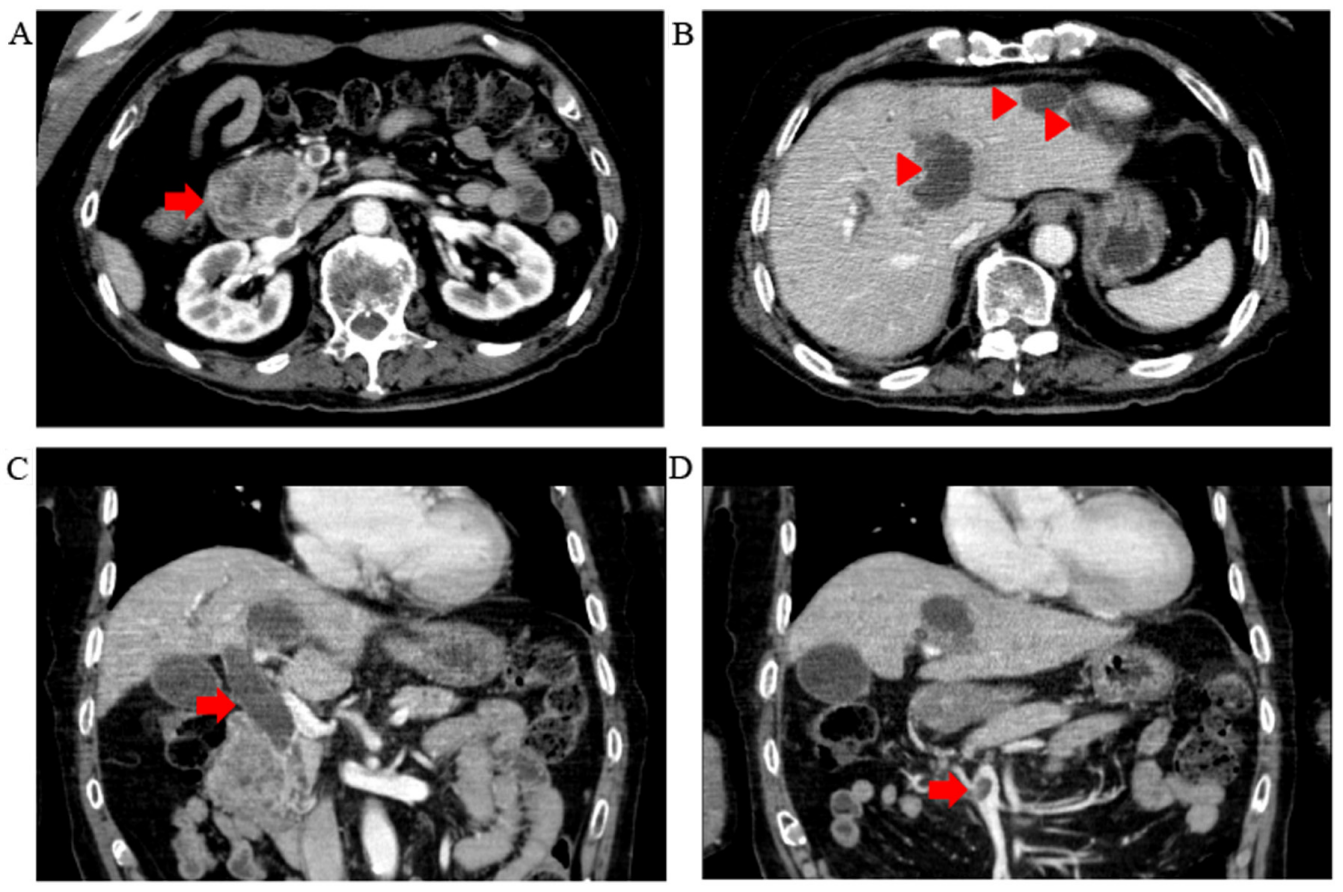
Abdominal contrast-enhanced CT at the patient's first visit. (A) A polycystic tumor was identified in the pancreatic head (arrow). (B) Polycystic low-density areas in the liver (arrowheads). (C) The pancreatic tumor obstructed the common bile duct, and the proximal part of the bile duct was dilated to 20 mm (arrow). (D) A 10-mm area with lack of enhancement was identified in the superior mesenteric vein (arrow).

**Figure 2:**
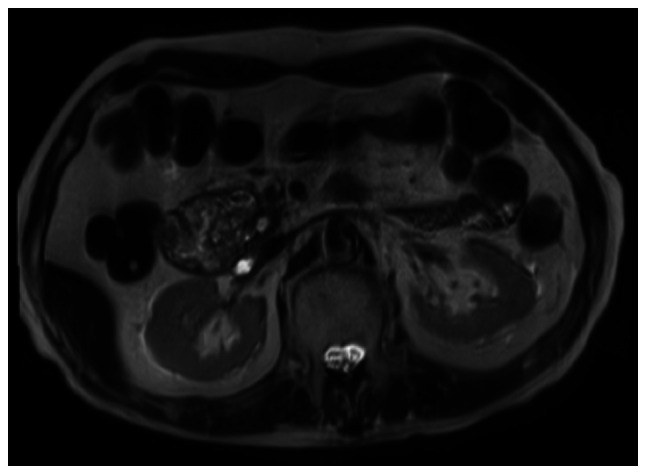
Magnetic resonance cholangiopancreatography signal from a T2-weighted image at the patient's first visit. The pancreatic head tumor exhibited low intensity, with a small high-intensity area.

**Figure 3:**
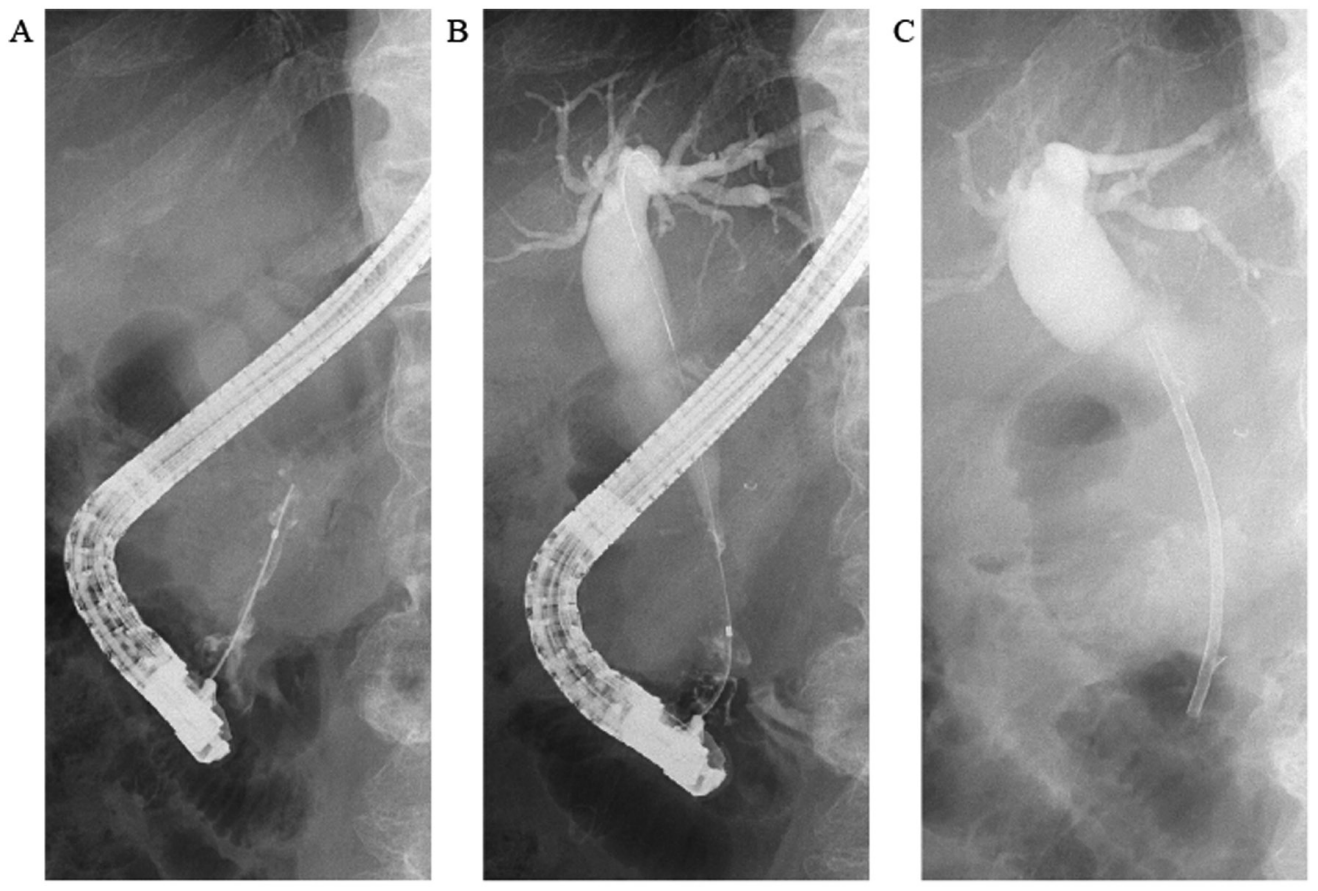
Endoscopic retrograde cholangiopancreatography was performed to drain his bile duct and enable diagnosis. (A) The main pancreatic duct was widened to 4 mm with mucus pooling. (B) The proximal bile duct was clearly widened, and the distal bile duct was narrowed over a length of 20 mm. (C) Endoscopic biliary stenting was performed using a plastic stent.

When the patient first visited our hospital in March 2018, his vital signs were stable, with a body temperature of 36.3˚C, heart rate of 56 bpm, blood pressure of 120/66 mmHg, respiratory rate of 16 breaths/min and SpO_2_ of 96% in room air. The conjunctivas were slightly anemic. There were no murmurs or rales on chest auscultation, and the abdomen was soft and flat with no tenderness on palpation. The laboratory tests (Table I) revealed mild normocytic anemia, reduced levels of albumin and increased levels of hepatic and biliary enzymes, including aspartate aminotransferase, alanine aminotransferase, alkaline phosphatase, γ-glutamyl transpeptidase and pancreatic amylase. The levels of tumor markers, including carbohydrate antigen (CA)19-9, DUPAN-2, carcinoembryonic antigen and α-fetoprotein were measured, and the CA19-9 level was found to be increased. To evaluate the pancreatic and liver masses, an abdominal contrast-enhanced CT scan was performed (Fig. 1). A polycystic low-density area was identified in the pancreatic head, with a width of 46 mm, and the wall of this polycystic area was partially well-enhanced. The common bile duct was obstructed due to the polycystic tumor in the pancreatic head and was dilated to 20 mm. Multiple polycystic low-density areas were identified in the liver, with a morphology similar to that of the cystic lesion in the pancreatic head. Moreover, a 10-mm filling defect was observed in the superior mesenteric vein (SMV). The magnetic resonance cholangiopancreatography signals from a T2-weighted image revealed that the pancreatic head tumor exhibited low intensity, with a small high-intensity area (Fig. 2). Similarly, on the CT scan, the common bile duct was obstructed by the polycystic tumor in the pancreatic head and was dilated to a width of 20 mm. In order to drain the bile duct and perform diagnostic brush cytology, endoscopic retrograde cholangiopancreatography (ERCP) was performed (Fig. 3). The papilla of Vater was normal. First, contrast medium was injected into the pancreatic duct. The main pancreatic duct was dilated to a width of 4 mm. Pooling of mucus in the pancreatic duct was suspected due to the shallow filling defects. Second, contrast medium was injected into the bile duct. The proximal bile duct was markedly dilated, whereas the distal bile duct was narrowed and was 20 mm in length. Brush cytology of the narrowed bile duct was conducted, and endoscopic biliary stenting (EBS) was performed using a plastic stent in order to drain the bile duct. The brush cytology findings revealed the presence of atypical ductal cells.

The comprehensive diagnosis was advanced IPMC with invasion of the bile duct and liver metastasis. The clinical stage was stage IV (T3N0M1) according to the Union for International Cancer Control TNM classification (6). As regards treatment, systemic chemotherapy was recommended; however, the patient and his family opted for best supportive care due to his declining performance status (performance status score: 4). Thus, EBS was performed to drain the bile duct on the 2^nd^ day of hospitalization and the plastic stent was exchanged for a metallic stent on the 8^th^ day to prolong stent patency. Subsequently, the patient developed cholangitis and aspiration pneumonia, and he eventually succumbed to IPMC 90 days after the initial admission.

**Figure 4:**
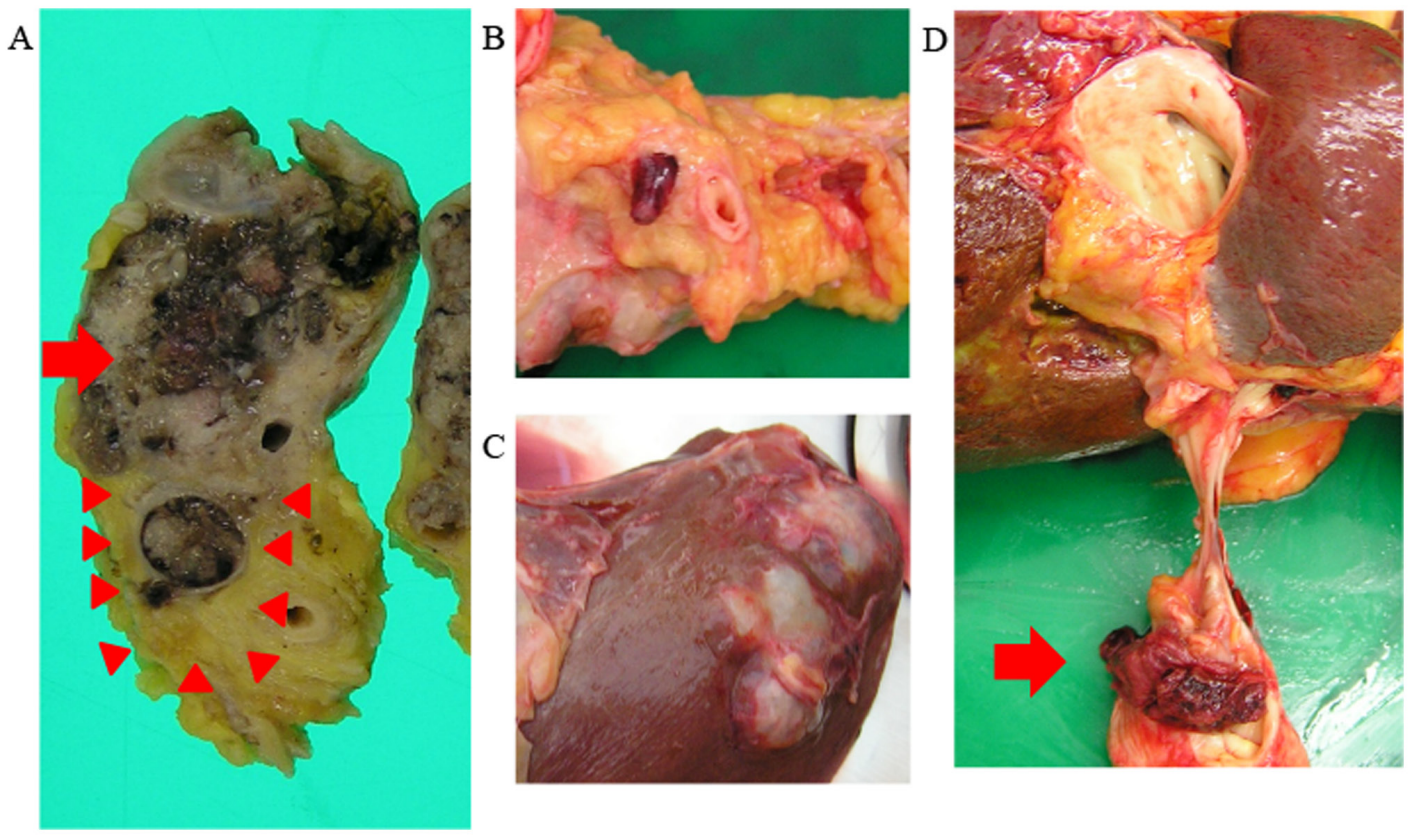
Macroscopic findings. (A) The pancreatic head tumor (arrow) invaded the portal vein (arrowheads). (B) SMV tumor thrombosis. (C) Metastatic liver lesion. (D) The tumor thrombus extended from the SMV to the hilar portal vein (arrow). SMV, superior mesenteric vein.

After obtaining informed consent from the patient's daughter, an autopsy was performed to examine the tumor. Macroscopically, the polycystic tumor in the pancreatic head was sized 5x4.5 cm and the cysts contained mucus. Moreover, the tumor had invaded the portal vein and formed an elongated tumor thrombus, extending from the SMV to the hilar portal vein. Polycystic tumor lesions were also identified in the liver, and the structure of these metastatic lesions was identical to that of the primary pancreatic head tumor (Fig. 4).

**Figure 5:**
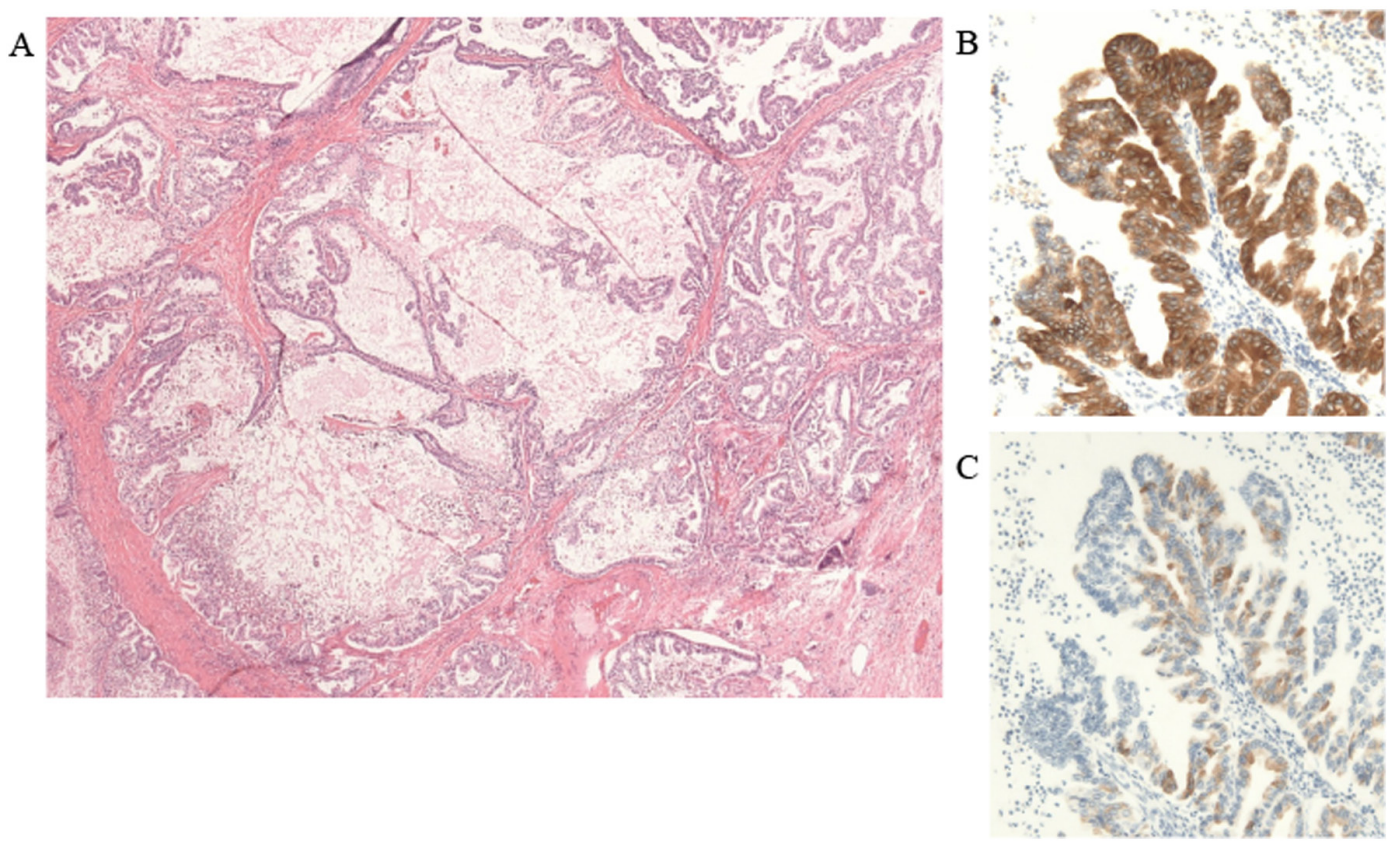
Microscopic findings of the pancreatic head lesion. (A) The tumor displayed a papillary structure consisting of small tumor cells. The cysts contained mucus (hematoxylin and eosin staining; magnification, x40). (B) Positive MUC1 staining (magnification, x100). (C) Slightly positive MUC5AC staining (magnification, x100). MUC, mucin core protein.

**Figure 6:**
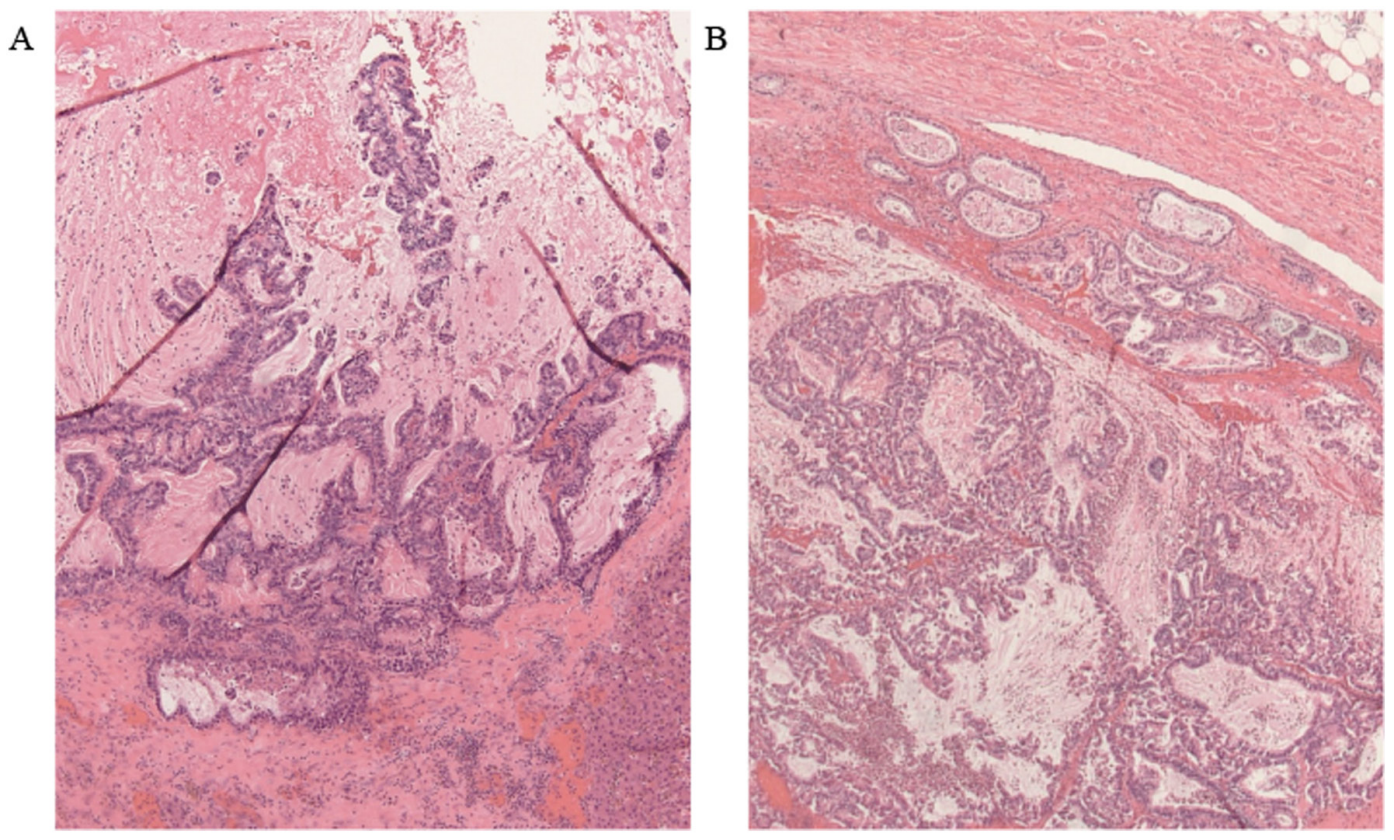
Microscopic findings of the liver metastatic lesion (hematoxylin and eosin staining; magnification, x40). (A) The polycystic liver tumors had the same structure as the pancreatic tumors on pathological examination. The polycystic lesions displayed a papillary arrangement with mucus pooling. (B) The structure of the superior mesenteric vein tumor thrombus was identical to that of the primary pancreatic tumor.

Microscopically, on hematoxylin and eosin staining, the pancreatic head tumor exhibited a papilliform structure consisting of small tumor cells and the tumor cysts were filled with mucus. On immunostaining, MUC1 and MUC5AC were positive (Fig. 5), but MUC2 was negative (data not shown). Therefore, the histological diagnosis was pancreatobiliary type IPMC, and the morphological diagnosis, which was based on the dilation of both the main pancreatic duct as well as its branches, was mixed-type IPMC. The polycystic liver tumors and SMV tumor thrombus had the same morphology as the pancreatic tumors on pathological examination, with pooling of mucus in the cysts of the liver metastatic lesions (Fig. 6). The pancreatic head tumor had not metastasized to the regional lymph nodes, but rather directly metastasized to the liver via the portal vein, retaining the IPMC structure and mucus production.

## Discussion

There were two clinical issues with the present case: First, the pancreatic tumor directly invaded the portal vein and caused tumor thrombosis. Second, the pancreatic tumor metastasized to the liver via the portal vein, retained the IPMC structure and produced mucus. Currently, invasive IPMN is considered to have a better prognosis compared with conventional PDAC (7,8). Ueda *et al* (9) reported the case of a female patient with IPMN with metastases to the lymph nodes, liver and lung, who achieved long-term survival (5 years and 2 months), as she responded to chemotherapy following pancreaticoduodenectomy. However, in the present case, the patient's survival duration after diagnosis was very short due to the widespread local invasion, distant metastases and lack of anticancer treatment, such as systemic chemotherapy or radiotherapy.

IPMN and other cystic pancreatic tumors may be accurately and timely diagnosed due to the advances in imaging techniques, including MRI and ERCP (10). On occasion, these tumors are incidentally found on abdominal CT in asymptomatic patients. Other patients may present with variable symptoms, such as abdominal pain, obstructive jaundice and vomiting, as in the present case. The progression of obstructive jaundice with IPMN has been previously reported, as mucus produced from IPMN may obstruct the common bile duct (11). However, in the present case, the common bile duct was directly invaded and obstructed by the tumor.

Serum CA19-9 level is a well-known predictor of PDAC and poor survival. Elevated serum CA19-9 levels were found to be associated with mixed invasive IPMC, which is consistent with several previous reports (12,13), and this finding may indicate that invasive IPMC shares similar characteristics with PDAC. Hirono *et al* (14) evaluated the diagnostic value of the factors associated with invasive IPMC identified in their study, and branch-duct IPMN with elevated serum CA19-9 levels was associated with invasive IPMC, with a sensitivity of 86.4%, specificity of 96.6%, positive predictive value of 86.4%, negative predictive value of 96.6% and accuracy of 94.5%.

IPMNs are classified into four subtypes (intestinal, pancreatobiliary, gastric and oncocytic) based on their histomorphology and mucin phenotype (15). The subtype of the present case was pancreatobiliary, as determined based on the positive expression of MUC1 and MUC5AC and negative expression of MUC2. The pancreatobiliary type is the most malignant among the four subtypes (5). Moreover, the main-duct or mixed-type morphological classifications of IPMN are associated with a higher risk of evolving into invasive IPMC compared with the branch-duct type (16). Mixed-type IPMN includes various histological types. Masuda *et al* (17) previously reported that mixed IPMN with positive MUC2 expression had a significantly higher prevalence of high-grade dysplasia and invasive IPMC compared with MUC2-negative IPMN. However, the tumor in the present case was classified as invasive IPMC, despite the negative MUC2 expression. It was previously reported that the mixed type of IPMN rarely includes the pancreatobiliary type (17). The pancreatobiliary type is MUC2-negative, as are the gastric and oncocytic types. Thus, this contradiction regarding whether mixed-type IPMN is invasive may be attributed to selection bias in the report.

It was reported that 19-45% of IPMNs are accompanied by invasive carcinomas (8). However, the presence of portal vein invasion was significantly lower among patients with invasive IPMC compared with those with PDAC (8). Invasive IPMCs with portal vein invasion and tumor thrombosis, such as in the present case, have rarely been reported (16,18). Moreover, our patient had a long tumor thrombus and liver metastases. When an abdominal contrast-enhanced CT was first performed, the tumor thrombus was only 10 mm. However, when the autopsy was performed after 90 days, the tumor thrombus had grown and extended from the SMV to the hilar portal vein. The rapid growth of the portal thrombosis indicated a highly malignant tumor. In addition, invasion into the vasculature is a signiﬁcant predictor of poor outcome (19). Moreover, in the present case, the structure of the polycystic liver tumors was identical to that of the pancreatic tumors on pathological examination. The SMV tumor thrombosis metastasized to the liver, maintaining its polycystic structure and mucus production. As the pancreatic head tumor had not metastasized to the regional lymph nodes, it was inferred that part of the SMV tumor thrombus, with a polycystic structure and mucus content, was detached and reached the liver via the portal vein.

While the patient and his family opted for best supportive care in the present case, several therapies for invasive IPMC have been reported. Pancreatectomy with lymph node dissection are recommended for invasive IPMC and PDAC, if the tumor is resectable (20), whereas the efficacy of postoperative adjuvant therapy for invasive IPMC remains controversial. McMillan *et al* (21) reported that postoperative adjuvant therapy may improve the survival of patients with advanced-stage invasive IPMC or lymph node metastases. However, another study reported that postoperative adjuvant therapy does not affect the survival of patients with invasive IPMC (22). Moreover, it is unclear whether systemic chemotherapy improves the survival of patients with unresectable invasive IPMN. Chemoradiotherapy has been demonstrated to prolong the survival of patients with PDAC (23). Regarding chemotherapy for PDAC, gemcitabine or S-1 (tegafur, gimeracil, oteracil potassium) as systemic chemotherapy for unresectable invasive IPMC is clinically considered (24).

To date, there have been reports of IPMC with portal invasion or liver metastasis (25,26). However, to the best of our knowledge, this is the first report of a IPMC metastasizing to the liver while maintaining its IPMC structure.

As regards the limitations of the present study, the expression of proliferating cell nuclear antigen, tumor protein p53, vascular endothelial growth factor and the KRAS mutation status were not determined due to health insurance-related restrictions. From an academic perspective, however, immunohistochemical examination must be performed to evaluate the status of carcinogenesis.

In conclusion, we herein present our experience with an autopsy case of IPMC accompanied by a long tumor thrombus in the portal vein and multifocal liver metastasis that maintained the structure of the primary pancreatic head tumor.

## Figures and Tables

**Table I:** Laboratory data at the patient's first visit.

Laboratory parameters (normal range)	Value	Unit
Hematology		
White blood cell count (3,300-8,600)	4,600	/µl
Red blood cell count (435-555x10^4^)	351x10^4^	/µl
Hematocrit (40.7-50.1)	32.1	%
Hemoglobin (13.7-16.8)	10.6	g/dl
Mean corpuscular volume (83.6-98.2)	91.5	fl
Platelet count (15.8-34.8x10^4^)	16.1x10^4^	/µl
Coagulation		
Prothrombin time-INR (0.85-1.15)	1.03	INR
Activated partial thromboplastin time (25-40)	31.9	sec
Fibrinogen (150-400)	491	mg/dl
Fibrin degradation products (0-10)	3.4	µg/ml
D-dimer (0-1)	1.0	µg/ml
Chemistry		
Albumin (4.1-5.1)	3.2	g/dl
Aspartate transaminase (13-30)	41	IU/l
Alanine transaminase (10-42)	66	IU/l
Lactate dehydrogenase (124-222)	86	IU/l
Alkaline phosphatase (106-322)	788	IU/l
γ-Glutamyl transpeptidase (13-64)	160	IU/l
Amylase (44-132)	357	IU/l
P-amylase (16-52)	282	IU/l
Total bilirubin (0.4-1.5)	0.5	mg/dl
Blood urea nitrogen (8-20)	16.5	mg/dl
Creatinine (0.65-1.07)	0.75	mg/dl
Na (138-145)	134	mEq/l
K (3.6-4.8)	4.2	mEq/l
C-reactive protein (0-0.29)	5.165	mg/dl
Procalcitonin (0-0.4)	0.39	ng/ml
Brain natriuretic peptide (0-18.4)	19.6	pg/ml
Hemoglobin A1c (4.7-6.2)	8.7	%
Tumor markers		
Carbohydrate antigen 19-9 (0-37)	84.6	U/ml
DUPAN-2 (0-150)	46	U/ml
Carcinoembryonic antigen (0-5)	3.5	ng/ml
α-Fetoprotein (0-15)	2.0	ng/ml
Protein induced by vitamin K absence-II (0-39)	25.45	mAU/ml

INR, international normalized ratio.

## Data Availability

All data generated or analyzed during this study are included in this published article.
